# Predictors of interruptions in antiretroviral therapy among people living with HIV in Nigeria: a retrospective cohort study using the Nigeria national data repository

**DOI:** 10.1371/journal.pone.0300937

**Published:** 2025-03-20

**Authors:** Sunday Ikakke Ikpe, Bashorun Ade, Aliyu Gambo, Rebecca Nowak, John D. Sorkin, Manhattan Charurat, Timothy O’Connor, Kristen Stafford

**Affiliations:** 1 University of Maryland Baltimore School of Medicine, Baltimore, Maryland, United States of America,; 2 Federal Ministry of Health, Nigeria,; 3 Nigeria AIDS Control Agency, Abuja, Nigeria,; 4 Baltimore V.A. Medical Center Geriatric Research, Education and Clinical Center,; 5 University of Maryland School of Medicine, Department of Medicine, Division of Gerontology, Geriatrics and Palliative Care; SKYDA Health Nigeria, NIGERIA

## Abstract

This study aimed to identify predictors of time to first interruption in treatment (IIT) and predictors of ever being interrupted in ART treatment among PLHIV in Nigeria using a national longitudinal dataset that covers all PEPFAR-funded implementing partners to inform national strategies to prevent IIT. This retrospective cohort study used data from Nigeria’s National Data Repository (NDR). The NDR is a de-identified longitudinal database of over 1.9 million PLHIV who received ART in Nigeria beginning in 2004 and is owned by the Federal Ministry of Health (FMoH). The NDR contains patient-level demographics, clinic visits, laboratory, and ART prescription and refill data uploaded at least monthly. The data extracted for this study were obtained from electronic medical record systems of 2,226 public facilities offering HIV care in the country. In this study, we investigated the predictors of treatment interruption using data from the national HIV treatment program. We identified sets of predictors of first interruption in treatment using the logistic regression and these to be consistent in predicting time to first interruption including sex, anchor drug in ART regimen, recorded HIV viral load, recorded CD4 cell count, WHO clinical staging, functional status, last measured weight, highest education attained, occupation, marital status, year enrolled in care, pre and post surge, pre and post-COVID and residing in a state capital, Lagos, or the federal capital territory (FCT) (capital city dweller) versus other locations (non capital city dweller). Age grouping was the only variable that was predictive only for time to first interruption but not for having a first interruption. To reduce the risk of IIT it is important to target interventions preemptively. We have highlighted the need for tailored interventions that address the unique needs of PLHIV in Nigeria. Targeted interventions focusing on those with a combination of risk factors could include education, counseling, supportive services, and monitoring and outreach.

## Introduction

Over 40 years since its emergence, human immunodeficiency virus (HIV) remains a significant public health concern. This is particularly true in sub-Saharan Africa, which bears 67% of the global burden^1–3^, where HIV remains one of the leading causes of death. [1] Antiretroviral therapy (ART) is critical for both individual-level management of HIV infection to preserve or improve health, and to prevent the onward transmission of the virus for epidemic control [2–4]. While tremendous progress has been made over the last 20 years with the support of the President’s Emergency Plan for AIDS Relief [5] and other international donors to expand access to ART [6], globally 15% of people do not know they are living with HIV, 25% are not on treatment, and 32% are not virally suppressed which inhibits progress towards HIV-1 epidemic control [1]. UNAIDS first announced global targets for progress toward epidemic control in 2014 and then updated them in 2020 [7]. The current goal, known as the 95-95-95 target, is for 95% of people living with HIV to be diagnosed, 95% of those who know their status to be on ART, and 95% of those on treatment to be virally suppressed [1]. ART is a lifelong treatment with no cure or vaccine available for HIV.

Based on the 2018 population-based Nigeria AIDS Impact and Indicator Survey, only 47% of people living with HIV have been diagnosed [8]. While the survey revealed that 96% of people who had been diagnosed were on ART, viral suppression among those on treatment was only 81% [9], meaning across the entire cascade only 37% of PLHIV in Nigeria were virally suppressed. Multiple factors may inhibit the achievement of viral suppression targets, as has been observed in Nigeria. These factors may be care or person-related [10]. Interruptions in treatment (IIT) pose a threat to achieving the expected gains of ART for PLHIV and their communities.

Prior studies have been carried out to understand the predictors of IIT and loss to follow-up (LTFU) in not just Nigeria, but in other sub-Saharan countries, including Mali, Ethiopia, and Rwanda, and have identified different sets of predictors including age, baseline CD4 count, sex, marital status, education, access to care, treatment regimens, and treatment fatigue to mention a few [11–17]. Most of the evidence generated from Nigeria has been based on cohorts from groups of health facilities supported by individual organizations that may employ specific models of care and can also limit geographic representation. These studies have employed only logistic regression or survival analysis as a means of identifying predictors of LTFU or IIT. While each approach is valid, each measures a different outcome. These differences may account for narrow and contradictory findings related to factors driving IIT and limit the generalizability of these studies to other regions [11,17,18].

This study aimed to identify predictors of the time to first IIT and predictors of ever being interrupted in ART treatment among PLHIV in Nigeria using a national longitudinal dataset that covers all PEPFAR-funded implementing partners to inform national strategies to prevent IIT.

## Methods

### Design and data source

This retrospective cohort study used medical records from Nigeria’s National Data Repository (NDR). The NDR is a de-identified longitudinal database of over 1.9 million PLHIV who received ART in Nigeria beginning in 2004 and is owned by the Federal Ministry of Health (FMoH) [16]. The NDR collects patient-level data from electronic medical records (EMRs) at HIV healthcare facilities across Nigeria. The data is encoded in extensible markup language (XML) and transmitted to the NDR via a secure hypertext transfer protocol (HTTPS). Data is sent weekly with the NDR collecting a wide range of data, including patient demographics, fingerprint data, HIV testing details, laboratory test results, treatment information, and other clinical encounter specifics. To maintain data quality, the NDR employs a robust validation process including XML file schema validation and data quality checks before being processed and stored. Files with errors are returned to the healthcare facilities for correction, promoting continuous data quality improvement. The NDR has observed a decrease in the proportion of rejected files over time, indicating improvements in data quality. Fingerprint template data is used to match, uniquely identify, and deduplicate patient records, enhancing the accuracy of treatment coverage assessments and program decision-making. The NDR has a low rate of duplicated records (less than 3%) [19]. Local approvals for the study were obtained from the National Health Research Ethics Committee of Nigeria (NHREC) with protocol number NHREC/01/01/2007-05/08/2024 and approval number NHREC/01/01/2007-13/08/2024. The data was obtained on the 1^st^ of November 2021 following a University of Maryland Baltimore (UMB) institutional review board (IRB) determination that the research is nonhuman subject research. At no point did we, the authors, have access to any form of personally identifiable information. The NDR contains patient-level demographics, clinic visits, laboratory, and ART prescription and refill data uploaded at least monthly. The data extracted for this study were obtained from electronic medical record systems of 2,226 of the 2,408 public facilities offering HIV care in the country and reporting to the NDR as of August 2021.

### Study population and variable definitions

The cohort was of adults (18 years and above) living with HIV in Nigeria who were enrolled in care at facilities reporting to the NDR, and who commenced HIV treatment from January 1 2015 to March 1, 2021, and followed to a maximum date of August 31, 2021. To be included in the analysis, patients had to have had at least two ART visits.

The outcome variable of interest in this study was the first interruption in treatment (IIT), defined as the first instance of no documented pharmacy refill within 28 days after the expiry of their last ART refill, followed by a return to treatment. The variable was derived from the difference between the last refill visit date and the next scheduled refill date which was then binary categorized. Variables of interest included age categories (18 – 24 years and subsequently in 10-year bins), sex (male, female), year of enrollment in care (2015 - 2021), local government area of care (capital city, non capital city), anchor drug in ART regimen, employment (employed, unemployed, student or retired), marital status, measured and recorded CD4, measured and recorded HIV viral load in copies per ml, WHO clinical staging (including an undocumented level as a proxy for quality of HIV care), functional status (bedridden, ambulatory, working), and last measured weight measured in kilograms. Additionally, a variable to assess the difference in IIT before and after the SARS-CoV-2 (COVID) pandemic lockdown of March 2020 was created using a cut point of March 2020 when there was a global lockdown. Similarly, a variable was created to assess the effect of a PEPFAR Surge initiative [20] to identify more people who were infected with HIV in Nigeria in 2019.

### Statistical analysis

The variables were analyzed considering the frequencies and proportions for categorical variables and the median and interquartile ranges at each of these levels for continuous variables. Chi-square and t-test p-values were reported. Logistic regression was used to estimate the odds of first treatment interruption. Determining potential predictors was based on p-values less than a threshold determined using the Bonferroni approach of dividing 0.05 by the number of variables in the final model. Odds ratios and 95% confidence intervals were estimated for the final selected predictors, along with p-values for these estimates. The outcome of survival analysis was the time to the first interruption event. All individuals who did not experience interruption were censored at the end of the follow-up period while those who met the definition of IIT were censored after the first event. Patients who did not return to care after the interruption event were assumed to have been lost to follow-up or dead and were excluded, restricting the analysis to IIT and patients who continue in care. A long rank test was used to assess differences in time to IIT generated using Kaplan-Meier plots for each categorical variable. Backward elimination was used to determine the variables to be included in a Cox proportional hazards regression model to produce hazard ratios and associated 95% confidence intervals.

The analysis was performed using R programming language version 4 and the survival package.

## Results

**Fig 1:**
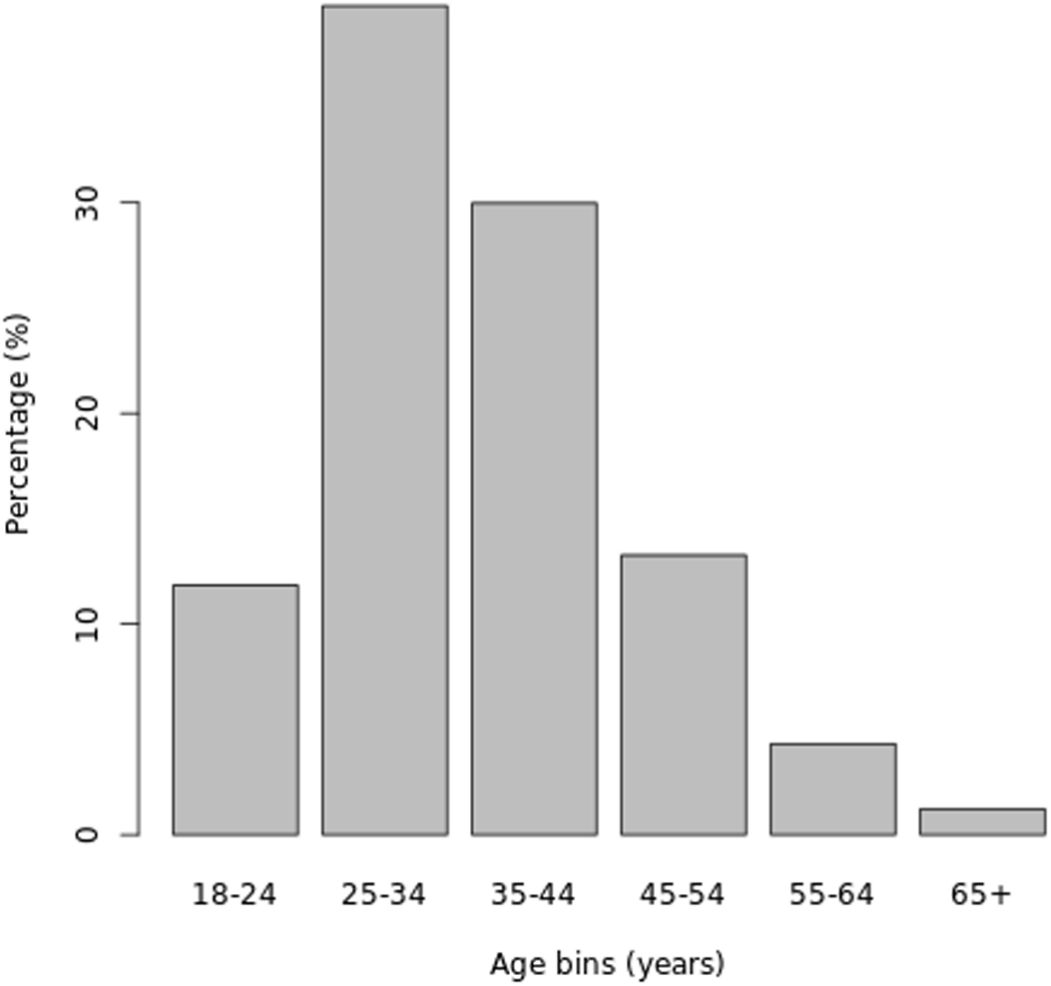
Distribution of age categories.

The NDR dataset contained records of 4,805,247 people, 32,758,238 clinic visits, 31,612,050 regimen records, and 24,931,718 laboratory results. After merging the datasets, 924,847 adults from 2,204 facilities were eligible for inclusion in the study. The records show an upward trend in enrollment from 89,535 in 2015 to 264,777 in 2020. The 2019 and 2020 surge years had the highest number of enrollees with 510,117(55.20%). The study sample was predominantly women 606,222(65.50%) and aged 25-34 years (Fig 1). The most commonly prescribed ART regimen included integrase strand transfer inhibitors (INSTI) 434,289(47.00%), followed by non-nucleoside reverse transcriptase inhibitors (NNRTI) 369,891(40.00%), protease inhibitors (PI) 5,624(0.60%) and nucleoside reverse transcriptase inhibitors (NRTI). There were 29,648(12.40%) records of undocumented ART. Less than a quarter of the study subjects (215,389, 23.3%) had a recorded CD4. Similarly, slightly over one-third (317,195, 34.3%) had a recorded HIV viral load.

During the study period, 500,729 (54.10%) patients experienced an IIT. Within the first month of care, 0.1% experienced their first interruption; within three months, this increased to 29.28%, and this further increased to 46.49% in the first six months and 50.26% in the first year. The distribution of participant characteristics between censored and interrupted observations is presented in Table 1. A higher proportion of women experienced interruptions, with 345,115 (56.93%) having an interruption compared to 155,614 (48.84%) males. Age was also associated with interruptions (p <  0.001). The proportion of interruptions increased progressively with increasing age categories 55,454 (50.16%) in the 18-24 years group and was highest in the 55-64 years group with 23,830 (50.58%) interruptions. The number of people aged 65 years and older who experienced IIT was 6,752 (59.11%).

**Table 1:** Variable characteristics in people who presented for a clinic visit.

	No Interruption in Treatment (N/%)	Interruption in Treatment (N/%)	P-value
**Sex**			<0.001
Women	261,107 (43.07%)	345,115 (56.93%)	
Men	163,011 (38.4%)	155,614 (48.84%)	
**Age Categories**			<0.001
18-24years	54,112 (49.39%)	55,454 (50.61%)	
25-34years	172,416 (47.39%)	191,383 (52.61%)	
35-44years	124,174 (44.78%)	153,107 (55.22%)	
45-54years	52,580 (42.82%)	70,203 (57.18%)	
55-64years	16,165 (40.42%)	23,830 (59.58%)	
65 + years	4,671 (40.89%)	6,752 (59.11%)	
**Anti retroviral therapy**			<0.001
INSTI	284,802(65.58%)	149,487 (29.90%)	
NNRTI	70,468 (19.05%)	299,423 (80.95%)	
PI	1,013 (18.01%)	4,611 (81.99%)	
Undocumented	67,835 (58.96%)	47,208 (41.04%)	
**CD4 recorded**			<0.001
No	378,398 (53.34%)	331,060 (46.66%)	
Yes	45,720 (21.23%)	169,669 (78.77%)	
**HIV Viral Load recorded**			<0.001
No	347,586 (57.20%)	260,066 (42.80%)	
Yes	76,532 (24.13%)	240,663 (75.87%)	
**WHO Clinical Stage**			<0.001
Unknown	19,368 (41.23%)	27,610 (58.77%)	
Stage 1	369,282 (49:17%)	381,787 (50.83%)	
Stage 2	20,651 (26.03%)	58,678 (73.97%)	
Stage 3	13,560 (30.81%)	30,448 (69.19%)	
Stage 4	1,257 (36.30%)	2,206 (63.70%)	
**Functional Status**			<0.001
Unknown	14,409 (31.99%)	30,632 (68.01%)	
Bedridden	1,082 (53.62%)	936 (46.38%)	
Ambulatory	2,278 (33.09%)	4,607 (66.91%)	
Working	406,349 (46.66%)	464,554 (53.34%)	
**Last measured weight** ^*^	58 (18)	57 (19)	<0.001
**Highest education attained**			<0.001
Unknown	198,716 (41.40%)	281,226 (58.60%)	
None	29,329 (47.49%)	32,429 (52.51%)	
Q’ranic	6,692 (33.79%)	13,115 (66.21%)	
Primary	41,198 (51.06%)	39,492 (48.94%)	
Secondary	120,869 (54.43%)	101,210 (45.57%)	
Tertiary	27,314 (45.09%)	33,257 (54.91%)	
**Occupation**			<0.001
Unknown	197,033 (42.52%)	266,355 (57.48%)	
Unemployed	127,083 (50.02%)	126,991 (49.98%)	
Student	14,035 (52.15%)	12,878 (47.85%)	
Employed	85,243 (8.37%)	93,403 (91.63%)	
Retired	724 (39.65%)	1,102 (60.35%)	
**Marital Status**			<0.001
Unknown	200,166 (41.71%)	279,713 (58.29%)	
Single	109,423 (58.63%)	77,221 (41.37%)	
Married	114,529 (44.34%)	143,794 (55.66%)	
Other	0 (0.00%)	1 (100.00%)	
**Year enrolled in care**			<0.001
2015	15,399 (17.20%)	74,136 (82.80%)	
2016	17,693 (16.58%)	89,044 (83.42%)	
2017	19,866 (17.60%)	93,006 (82.40%)	
2018	25,291 (23.95%)	80,295 (76.05%)	
2019	55,781 (37.59%)	92,601 (62.41%)	
2020	199,897 (74.10%)	69,880 (25.90%)	
2021	90,191 (98.08%)	1,767 (1.92%)	
**Surge**			<0.001
Pre-Surge	78,249 (18.87%)	336,481 (81.13%)	
Post-Surge	345,869 (67.80%)	164,248 (32.20%)	
**Enrolled after COVID**			<0.001
No	75,351 (16.63%)	377,656 (83.37%)	
Yes	348,767 (73.92%)	123,073 (26.08%)	
**Capital city vs non capital city**			<0.001
Capital city dweller	340,328 (45.04%)	415,230 (54.96%)	
Non capital city dweller	83,790 (49.50%)	85,499 (50.50%)	
**Hospital Encounters** ^*^	3 (3)	2 (2)	<0.001
**Time to interruption event (months)** ^*^	7 (9)	9 (11)	<0.001

*Median(Interquartile range) reported.

*NNRTI: Non nucleoside reverse transcriptase inhibitor*

*PI: Protease inhibitor*

*INST: Integrase strand transfer inhibitors*

**Fig 2:**
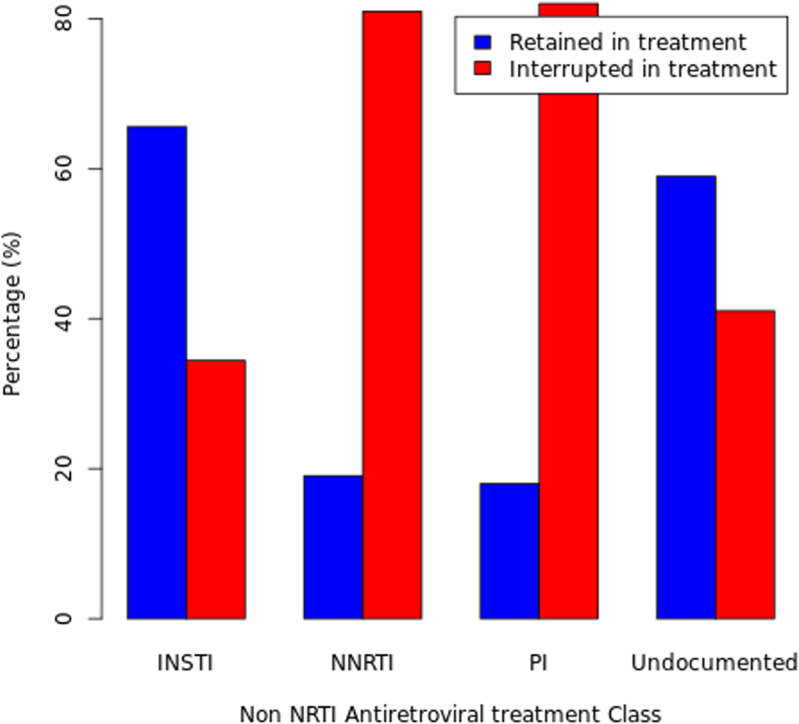
Distribution of Time to first IIT event by Antiretroviral medication.

Anchor drugs in the antiretroviral therapy (ART) regimen were associated with interruptions (p-value <  0.001). Among the different regimens, individuals on the INSTI had the highest proportion of retention in care (Fig 2), with 284,803 (65.58%). The NNRTIs had the highest interruption rate of 299,423(80.95%). PIs had 4,611(81.99%) interruptions, while undocumented regimens had 67,835(58.96%) interruptions. Those with recorded CD4 levels had a higher proportion of interruptions (169,669; 78.77%) compared to 46.66% for those with no records of CD4 count, similarly, those with recorded viral load had higher interruptions (240,663; 75.87%) compared to 42.80% for those with no recorded HIV viral load. The proportion with ambulatory functional status was most likely to experience interruption with 6.885(66.91%) experiencing interruption when compared to those who were bedridden (936,(46.38%)) or working (464554,(53.34%)).

PLHIV with primary and secondary education had lower proportions of interruption: 39,492(48.94%) and 101,210(45.57%), respectively while no education, qur’anic, and tertiary education had higher proportions of interruption: 32,429(52.51), 13,115(66.21%), and 33,257(54.91%), respectively. Employed people had the highest proportion of interruptions compared with other occupation groups, with 933,403(91.63%) experiencing interruption. This was followed by 1,102(60.35%) retirees. Students and unemployed had lower proportions of interruptions with 12,878(47.85%) and 126,991(49.98%), respectively.

Those enrolled in 2015, 2016, and 2017 had a higher proportion of interruptions, with 74,136 (82.80%), 89,044 (83.42%), and 93,006 (82.40%) interruptions, respectively. A higher proportion of interruptions occurred in patients who enrolled pre-surge (336,481, 81.13%) and pre-COVID (75,351; 83.87%).

The median number of hospital encounters differed between those who continued in care (3, IQR =  (2,5)) and those interrupted (2, IQR =  (2,4)) observations (p <  0.001). On average patients with interrupted observations had fewer hospital visits. Patients with interrupted observations experienced a longer time to interruption (median: 7 months; IQR: (3,9)) event than those who continued receiving ART uninterrupted (median: 9 months, IQR:(5,16)) without interruption.

### Logistic regression of interruption to treatment

The results of the statistical analysis are presented in Table 2; sex was associated with the outcome, with men having 0.90 (0.89, 0.91) times lower odds of IIT compared to women. Compared to people aged 25-34 years, there were no differences in the odds of experiencing IIT for those aged 18-24 years (aOR =  0.98, 95% CI: 0.96-1.00, p =  0.074), 35-44 years (aOR =  1.03, 95% CI: 1.01-1.04, p =  0.002), 45-54 years (aOR =  1.02, 95% CI: 0.99-1.04, p =  0.136), and 55-64 years (aOR =  1.02, 95% CI: 0.98-1.04, p =  0.321) and in the 65 years and above group (aOR =  0.96, 95% CI: 0.90-1.02, p =  0.147).

**Table 2:** Factors associated with interruption in HIV treatment among people living with HIV in Nigeria, 2015-2021.

	Adjusted Odds Ratios (aOR)	95% Confidence Interval	p-value
**Male sex (Ref. Women)**	0.90	0.89 – 0.91	<0.001
**Age Category (Ref. 25-34years)**			
18-24 years	0.98	0.96 – 1.00	0.074
35-44 years	1.03	1.01 – 1.04	0.002
45-54 years	1.02	0.99 – 1.04	0.136
55-64 years	1.02	0.98 – 1.05	0.321
65 + years	0.96	0.90 – 1.02	0.147
**ART Regimen (Ref. INSTI)**			
NNRTI	1.33	1.30 – 1.36	<0.001
PI	1.09	0.97 – 1.23	0.161
Undocumented	1.02	0.99 – 1.06	<.0.001
**Viral Load Recorded (Ref: Not recorded)**	3.12	3.07 – 3.16	<0.001
**CD4 Recorded (Ref. Not recorded)**	1.03	1.01 – 1.05	<0.001
**WHO clinical staging (Ref. Stage 1)**			
Stage 2	0.99	0.97 – 1.02	<0.001
Stage 3	0.55	0.54 – 0.57	<0.001
Stage 4	0.39	0.35 – 0.43	<0.001
Undocumented	--	--	--
**Functional Status (Ref. Working)**			
Ambulatory	0.36	0.35 – 0.38	<0.001
Bedridden	0.49	0.42 – 0.57	<0.001
Unknown functional status	0.59	0.55 – 0.63	<0.001
**Last measured Weight**	1.00	1.00 – 1.00	<0.001
**Highest Education (Ref. Secondary)**			
No education	1.01	0.97 – 1.04	0.724
Qur’anic	0.90	0.88 – 0.93	<0.001
Primary	0.97	0.92 – 1.02	0.242
Tertiary	0.82	0.79 – 0.84	<0.001
Unknown	1.04	1.01 – 1.07	0.017
**Occupation (Ref. Employed)**			
Retired	0.93	0.90 – 0.96	<0.001
Student	1.12	1.07 – 1.17	<0.001
Unemployed	0.94	0.91 – 0.97	<0.001
Unknown	0.91	0.79 – 1.05	0.206
**Marital Status (Ref. Married)**			
Single	0.96	0.93 – 0.98	0.001
Unknown	--	--	--
**Year Enrolled**	0.70	0.69 – 0.70	<0.001
**Surge Enrollee(Ref. Pre Surge enrollee)**	1.48	1.43 – 1.52	<0.001
**COVID Enrollee (Ref. Pre COVID enrollee)**	0.19	0.19 – 0.19	<0.001
**Capital city dweller (Ref. Non Capital city dweller)**	0.83	0.80 – 0.85	<0.001

*NNRTI: Non nucleoside reverse transcriptase inhibitor*

*PI: Protease inhibitor*

*INST: Integrase strand transfer inhibitors*

The type of anchor drug used in the antiretroviral (ART) regimen was associated with IIT. Compared to individuals on the INSTI regimen, those on NNRTI regimen had higher odds of the outcome (aOR =  1.33, 95% CI: 1.30-1.36, p =  < 0.001. Individuals on PI and undocumented regimens showed no significant difference in adjusted odds ratios of 1.09 (0.97, 1.23) and 1.02 (0.99, 1.06) respectively. People with a recorded viral load had higher odds of the outcome compared to those without a recorded viral load (aOR =  3.12, 95% CI: 3.07-3.16, p <  0.001) as with, to a lesser degree, people with recorded CD4 levels (aOR =  1.03, 95% CI: 1.01-1.05, p <  0.001).

People with a WHO Stage of II(aOR =  0.99, 95% CI: 0.97-1.02, p <  0.001), Stage III (aOR =  0.55, 95% CI: 0.54-0.57, p <  0.001), and Stage IV (aOR =  0.39, 95% CI: 0.35-0.43, p <  0.001) had lower odds of IIT compared to those in Stage I. Being ambulatory (aOR =  0.36, 95% CI: 0.35-0.38, p <  0.001) or bedridden (aOR =  0.49, 95% CI: 0.42-0.57, p <  0.001) had lower odds of the outcome compared to those classified as working.

Using secondary school education as a reference, no education (aOR =  1.01, 95% CI: 0.97-1.04, p =  0.724), primary education (aOR =  0.97, 95% CI: 0.92-1.02, p =  0.242) or undocumented education (aOR =  1.04, 95% CI: 1.01-1.07, p =  0.017) were not associated with IIT. Qur’anic education (aOR =  0.90, 95% CI: 0.88-0.93, p <  0.001), and tertiary education (aOR =  0.82, 95% CI: 0.79-0.84, p <  0.001) were associated with lower odds of the outcome. Retired (aOR =  0.93, 95% CI: 0.90-0.96, p <  0.001), and unemployed (aOR =  0.94, 95% CI: 0.91-0.97, p <  0.001) individuals had lower odds of the outcome compared to employed individuals. Students (aOR =  1.12, 95% CI: 1.07-1.17, p <  0.001) had higher odds of IIT compared to employed individuals. Having undocumented education was not associated with IIT (aOR = 0.91, 95%CI:0.79-1.05). Being single was protective against IIT (aOR =  0.96, 95% CI: 0.93-0.98, p <  0.001) compared to those who were married.

The year PLHIV were enrolled in treatment was associated with the outcome, with lower odds of the outcome for more recent enrollments (aOR =  0.70, 95% CI: 0.69-0.70, p <  0.001). Starting ART during the national surge efforts was associated with higher odds of IIT compared to the pre-surge period (aOR =  1.48, 95% CI: 1.43-1.52, p <  0.001), and enrollment during the early phase of the COVID-19 pandemic was associated with lower odds of IIT compared to the pre-COVID-19 period (aOR =  0.19, 95% CI: 0.19-0.19, p <  0.001). Residing in an capital city was associated with lower odds of the outcome than residing in a non capital city area (aOR =  0.83, 95% CI: 0.80-0.85, p <  0.001).

### Survival analysis of the first interruption in care of PLHIV

**Fig 3:**
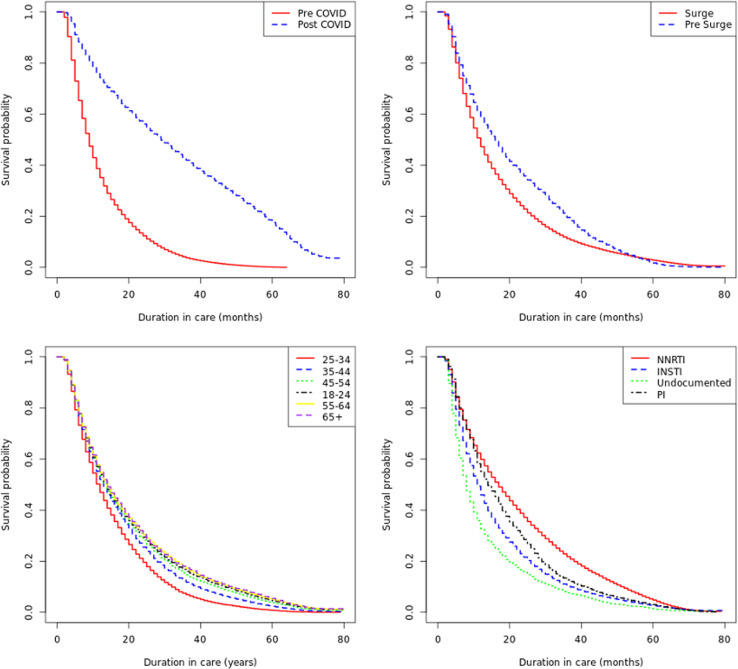
Kaplan Meier plots showing time to first interruption in ART versus enrollment pre/post surge commencement; pre/post-COVID pandemic; Antiretroviral therapy; and Age categories.

The initial exploration of the categorical variables using a long rank test and Kaplan-Meier plots (Fig 3). All categorical variables were statistically different in the life test with p-values < 0.001. The results from the multivariable analysis using Cox proportional hazards regression are presented in Table 3, showing the adjusted hazard ratios (aHR) along with their associated 95% confidence intervals (CI) and p-values for each variable. Sex was not associated with the outcome, with men having an adjusted hazard ratio of 1.00 (95% CI: 0.99-1.01) compared to women (p =  0.938). PLHIV aged 18-24 years had a 1.16 (1.14, 1.17) times higher hazard of the outcome compared to those aged 25-34 years. Conversely, those aged 35-44 years (aHR =  0.92, 95% CI: 0.91-0.93, p <  0.001), 45-54 years (aHR =  0.91, 95% CI: 0.90-0.92, p <  0.001), 55-64 years (aHR =  0.89, 95% CI: 0.88-0.90, p <  0.001), and 65 years and above (aHR =  0.89, 95% CI: 0.86-0.92, p <  0.001) had lower hazards of the outcome.

**Table 3:** Factors associated with time to first interruption in HIV treatment among people living with HIV in Nigeria, 2015-2021.

	Adjusted Hazard Ratios (aHR)	95% Confidence Interval	P-value
**Male sex (Ref: Women)**	1.00	0.99 – 1.01	0.938
**Age Category (Ref. 25-34years)**			
18-24 years	1.16	1.14 – 1.17	<0.001
35-44 years	0.92	0.91 – 0.93	<0.001
45-54 years	0.91	0.90 – 0.92	<0.001
55-64 years	0.89	0.88 – 0.90	<0.001
65 + years	0.89	0.86 – 0.92	<0.001
**ART Regimen (Ref. INSTI)**			
NNRTI	0.80	0.80 – 0.81	<0.001
PI	1.07	1.03 – 1.11	<0.001
Undocumented	0.90	0.88 – 0.92	<0.001
**Viral Load Recorded (Ref: Not recorded)**	1.05	1.04 – 1.05	<0.001
**CD4 Recorded (Ref. Not recorded)**	0.89	0.88 – 0.89	<0.001
**WHO clinical staging (Ref. Stage 1)**			
Stage 2	1.19	1.18 – 1.20	<0.001
Stage 3	1.27	1.26 – 1.29	<0.001
Stage 4	1.26	1.20 – 1.33	<0.001
Missing	1.31	1.29 – 1.34	<0.001
**Functional Status (Ref. Working)**			
Ambulatory	0.90	0.88 – 0.92	<0.001
Bedridden	1.09	1.00 – 1.19	0.040
Unknown functional status	1.09	1.05 – 1.13	<0.001
**Last measured Weight**	1.00	1.00 – 1.00	<0.001
**Highest Education (Ref. Secondary)**			
No education	1.00	0.99 – 1.02	0.963
Q’ranic	0.98	0.97 – 0.99	0.001
Primary	1.01	0.98 – 1.03	0.666
Tertiary	0.92	0.91 – 0.94	<0.001
Unknown	0.99	0.98 – 1.01	0.311
**Occupation (Ref. Employed)**			
Retired	1.01	0.99 – 1.02	0.351
Student	1.01	0.98 – 1.03	0.625
Unemployed	1.04	1.03 – 1.06	0.005
Unknown	1.10	1.03 – 1.17	0.012
**Marital Status (Ref. Married)**			
Single	0.98	0.97 – 1.00	0.007
Unknown	--	--	--
**Year Enrolled**	1.21	1.20 – 1.21	<0.001
**Surge Enrollee(Ref. Pre Surge enrollee)**	1.14	1.12 – 1.15	<0.001
**COVID Enrollee (Ref. Pre COVID enrollee)**	0.17	0.17 – 0.17	<0.001
**Capital city dweller (Ref. Non capital city dweller)**	1.06	1.05 – 1.07	<0.001

*NNRTI: Non nucleoside reverse transcriptase inhibitor*

*PI: Protease inhibitor*

*INST: Integrase strand transfer inhibitors*

Compared to individuals on the INSTI regimen, those on NNRTI (aHR =  0.80, 95% CI: 0.80-0.81, p <  0.001), and undocumented (aHR =  0.90, 95% CI: 0.88-0.92, p <  0.001) had a lower risk of interruptions in treatment within the same time period. Those on PI (aHR =  1.07, 95% CI: 1.03-1.11, p <  0.001) had a higher risk for interruption within the same time period when compared to those on INSTI. Patients with a recorded viral load had a 1.05 (1.04, 1.05) times higher instantaneous risk of the outcome compared to those without a recorded viral load. Those with recorded CD4 levels had a lower risk 0.89 (0.88, 0.89) than those without recorded CD4 levels within the same time period (Table 4).

**Table 4:** Characteristics of Month of interruption and state (location) by interruption status.

	No interruption in treatment	Interruption in treatment	p-value
**Month of interruption**			
January	10865 (2.6%)	50287 (10.0%)	<0.001
February	29541 (7.0%)	45649 (9.1%)	
March	42279 (10.0%)	50070 (10.0%)	
April	52780 (12.4%)	34052 (6.8%)	
May	90575 (21.4%)	35381 (7.1%)	
June	88122 (20.8%)	39169 (7.8%)	
July	56814 (13.4%)	35408 (7.1%)	
August	15981 (3.8%)	39425 (7.9%)	
September	9949 (2.3%)	41106 (8.2%)	
October	9766 (2.3%)	40996 (8.2%)	
November	8951 (2.1%)	45980 (9.2%)	
December	8495 (2.0%)	43206 (8.6%)	
**State**			
Abia	1758 (0.4%)	1678 (0.3%)	<0.001
Adamawa	7243 (1.7%)	12490 (2.5%)	
Akwa Ibom	82318 (19.4%)	54054 (10.8%)	
Anambra	4692 (1.1%)	13244 (2.6%)	
Bauchi	6172 (1.5%)	9539 (1.9%)	
Bayelsa	4271 (1.0%)	4026 (0.8%)	
Benue	32416 (7.6%)	31135 (6.2%)	
Borno	3028 (0.7%)	5533 (1.1%)	
Cross River	16588 (3.9%)	24123 (4.8%)	
Delta	21473 (5.1%)	9217 (1.8%)	
Ebonyi	960 (0.2%)	898 (0.2%)	
Edo	3224 (0.8%)	8567 (1.7%)	
Ekiti	563 (0.1%)	356 (0.1%)	
Enugu	7478 (1.8%)	3744 (0.7%)	
FCT	22395 (5.3%)	14832 (3.0%)	
Gombe	3605 (0.9%)	4104 (0.8%)	
Imo	4878 (1.2%)	6224 (1.2%)	
Jigawa	1457 (0.3%)	3453 (0.7%)	
Kaduna	4393 (1.0%)	4614 (0.9%)	
Kano	4217 (1.0%)	12724 (2.5%)	
Katsina	1918 (0.5%)	2465 (0.5%)	
Kebbi	2940 (0.7%)	4882 (1.0%)	
Kogi	3655 (0.9%)	4760 (1.0%)	
Kwara	1589 (0.4%)	3008 (0.6%)	
Lagos	34119 (8.0%)	34517 (6.9%)	
Nasarawa	12254 (2.9%)	11043 (2.2%)	
Niger	6481 (1.5%)	10698 (2.1%)	
Not recorded	47950 (11.3%)	123001 (24.6%)	
Ogun	3702 (0.9%)	7145 (1.4%)	
Ondo	1856 (0.4%)	1443 (0.3%)	
Osun	1068 (0.3%)	1187 (0.2%)	
Oyo	2750 (0.6%)	7664 (1.5%)	
Plateau	3222 (0.8%)	7092 (1.4%)	
Rivers	58358 (13.8%)	38431 (7.7%)	
Sokoto	1342 (0.3%)	3039 (0.6%)	
Taraba	4655 (1.1%)	10702 (2.1%)	
Yobe	1912 (0.5%)	3125 (0.6%)	
Zamfara	1218 (0.3%)	1972 (0.4%)	

Individuals with Stage II (aHR =  1.19, 95% CI: 1.18-1.20, p <  0.001), Stage III (aHR =  1.27, 95% CI: 1.26-1.29, p <  0.001), Stage IV (aHR =  1.26, 95% CI: 1.20-1.33, p <  0.001), and those with missing staging information (aHR =  1.31, 95% CI: 1.29-1.34, p <  0.001) had a higher risk of first interruption within the same time period compared to those in Stage I. Ambulatory patients (aHR =  0.90, 95% CI: 0.88-0.92, p <  0.001) had a lower instantaneous risk of the outcome, while being bedridden (aHR =  1.09, 95% CI: 1.00-1.19, p =  0.040) was not associated with the outcome. Those with unknown functional status (aHR =  1.00, 95% CI: 1.00-1.00, p <  0.001) were not associated with the outcome (Table 5).

**Table 5:** Adjusted odds ratios for month of interruption and state where interruption occurred.

	Adjusted odds ratios (aOR)	95% Confidence Intervals LCB	p-value
**Month of interruption (Ref=January)**			
February	0.44	0.43 - 0.46	<.001
March	0.38	0.36 - 0.39	<.001
April	0.25	0.24 - 0.26	<.001
May	0.17	0.16 - 0.18	<.001
June	0.21	0.20 - 0.22	<.001
July	0.23	0.22 - 0.23	<.001
August	0.62	0.60 - 0.65	<.001
September	1.03	0.98 - 1.07	0.226
October	0.78	0.74 - 0.81	<.001
November	1.16	1.11 - 1.21	<.001
December	1.35	1.29 - 1.41	<.001
**State (Ref=Akwa Ibom)**			
Abia	1.36	1.24 - 1.49	<.001
Adamawa	1.36	1.27 - 1.45	<.001
Anambra	4.1	3.91 - 4.30	<.001
Bauchi	3.5	3.29 - 3.73	<.001
Bayelsa	1.98	1.86 - 2.10	<.001
Benue	1.43	1.38 - 1.47	<.001
Borno	1.5	1.40 - 1.60	<.001
Cross River	1.95	1.87 - 2.02	<.001
Delta	1.01	0.97 - 1.05	0.664
Ebonyi	1.41	1.26 - 1.58	<.001
Edo	3.35	3.17 - 3.54	<.001
Ekiti	1.19	0.99 - 1.42	0.064
Enugu	2.36	2.24 - 2.49	<.001
FCT	1.7	1.63 - 1.77	<.001
Gombe	1.51	1.42 - 1.61	<.001
Imo	3.28	3.11 - 3.47	<.001
Jigawa	2.33	2.12 - 2.56	<.001
Kaduna	1.25	1.18 - 1.33	<.001
Kano	2.98	2.82 - 3.13	<.001
Katsina	1.22	1.12 - 1.32	<.001
Kebbi	2.99	2.79 - 3.21	<.001
Kogi	1.5	1.40 - 1.60	<.001
Kwara	2.97	2.73 - 3.23	<.001
Lagos	2.09	2.01 - 2.17	<.001
Nasarawa	1.48	1.42 - 1.54	<.001
Niger	2.67	2.53 - 2.82	<.001
Ogun	2.75	2.59 - 2.92	<.001
Ondo	1.02	0.93 - 1.13	0.678
Osun	1.96	1.76 - 2.19	<.001
Oyo	2.49	2.32 - 2.67	<.001
Plateau	1.29	1.20 - 1.39	<.001
Rivers	2.17	2.12 - 2.23	<.001
Sokoto	3.05	2.78 - 3.33	<.001
Taraba	1.9	1.81 - 1.99	<.001
Yobe	1.6	1.47 - 1.74	<.001
Zamfara	1.88	1.7 - 2.08	<.001

Logistic regression model: IIT =  LW +  VL +  CD4 +  Ambulatory +  Bedridden +  Unknown_Funcional_Status +  Male +  No_Education +  Tertiary_Education +  Primary_Education +  Qranic_Education +  Unknown_Edcuation +  Retired +  Student +  Unemployed +  Unknown_Employment +  Other_Marital_Status +  Single +  Unknown_Marital_Status +  Year_Enrolled_in_Care +  Surge +  COVID +  February +  March +  April +  May +  June +  July +  August +  September +  October +  November +  December +  Capital_City +  Abia +  Adamawa +  Anambra +  Bauchi +  Bayelsa +  Benue +  Borno +  Cross_River +  Delta +  Ebonyi +  Edo +  Ekiti +  Enugu +  Federal_Capital_Territoty +  Gombe +  Imo +  Jigawa +  Kaduna +  Kano +  Katsina +  Kebbi +  Kogi +  Kwara +  Lagos +  Nasarawa +  Niger +  Ogun +  Ondo +  Osun +  Oyo +  Plateau +  Rivers +  Sokoto +  Taraba +  Yobe +  Zamfara +  Age18_24 +  Age35_44 +  Age45_54 +  Age55_64 +  Age65ge +  NNRTI +  Undocumented_ART +  PI +  WHO_missing +  WHO_II +  WHO_III +  WHO_IV

Being uneducated (aHR =  0.1.00, 95% CI: 0.99-1.02, p <  0.001), having primary education (aHR =  1.01, 95%CI: 0.98-1.03), and undocumented education (aHR =  0.99, 95%CI: 0.98-1.01) were not associated with instantaneous risk of first interruption in treatment. Qur’anic education (aHR =  0.98, 95% CI: 0.97-0.99, p =  0.001), and tertiary education (aHR =  0.92, 95%CI: 0.91-0.94) were associated with a lower instantaneous risk of being interrupted compared to secondary education. When comparing being employed with retired (aHR =  1.01, 95% CI: 0.99-1.02), a student (aHR =  1.01, 95% CI: 0.98-1.03), unemployed (aHR =  1.04, 95%CI: 1.03-1.06), and undocumented occupation (aHR =  1.10, 95%CI: 1.03-1.17) did not show any association. Being single was not associated with an instantaneous risk of treatment interruption when compared to being married (aHR =  0.98, 95% CI: 0.97-1.00, p =  0.007) (Table 6).

**Table 6:** Adjusted hazard ratios (aHR) for month of interruption and state (location).

	Adjusted hazard ratios (aHR)	95% confidence intervals	p.value
**Month of interruption (Reference = January)**			
February	0.9	0.88 - 0.91	<.001
March	0.79	0.78 - 0.80	<.001
April	0.86	0.85 - 0.88	<.001
May	0.7	0.69 - 0.72	<.001
June	0.76	0.75 - 0.77	<.001
July	0.83	0.82 - 0.85	<.001
August	1.18	1.16 - 1.19	<.001
September	1.2	1.18 - 1.22	<.001
October	1.11	1.09 - 1.12	<.001
November	1.22	1.20 - 1.24	<.001
December	1.19	1.17 - 1.21	<.001
**State (Reference=Akwa Ibom)**			
Abia	0.74	0.71 - 0.78	<.001
Adamawa	0.95	0.92 - 0.98	0.001
Anambra	0.98	0.96 – 1.00	0.106
Bauchi	1.52	1.48 - 1.55	<.001
Bayelsa	1.03	1.00 - 1.07	0.075
Benue	0.85	0.83 - 0.86	<.001
Borno	1.11	1.07 - 1.14	<.001
Cross River	1.09	1.07 - 1.11	<.001
Delta	0.78	0.76 - 0.80	<.001
Ebonyi	1.26	1.18 - 1.35	<.001
Edo	1.37	1.34 - 1.40	<.001
Ekiti	0.81	0.73 - 0.90	<.001
Enugu	1.25	1.21 - 1.30	<.001
FCT	0.81	0.79 - 0.82	<.001
Gombe	0.87	0.84 - 0.90	<.001
Imo	1.37	1.33 - 1.41	<.001
Jigawa	1.35	1.30 - 1.40	<.001
Kaduna	1.21	1.17 - 1.25	<.001
Kano	1.16	1.14 - 1.19	<.001
Katsina	0.71	0.68 - 0.74	<.001
Kebbi	0.58	0.56 - 0.59	<.001
Kogi	1.05	1.02 - 1.08	0.002
Kwara	0.79	0.76 - 0.82	<.001
Lagos	0.96	0.94 - 0.98	<.001
Nasarawa	0.97	0.95 - 0.99	0.015
Niger	0.81	0.79 - 0.83	<.001
Ogun	1.07	1.04 - 1.10	<.001
Ondo	0.77	0.73 - 0.82	<.001
Osun	0.93	0.87 - 0.98	0.014
Oyo	1.36	1.32 - 1.40	<.001
Plateau	1.23	1.20 - 1.27	<.001
Rivers	1.02	1.01 - 1.04	0.008
Sokoto	0.99	0.95 - 1.02	0.491
Taraba	0.55	0.54 - 0.56	<.001
Yobe	1.13	1.09 - 1.18	<.001
Zamfara	0.68	0.65 - 0.71	<.001

Cox proportional hazards model: Time to IIT event =  Last_Redorded_Weight +  Viral_Load_Recorded +  CD4_Recorded +  Ambulatory +  Bedridden +  Unknown_Fucntional_Status +  Male +  No_Education +  Tertiary +  Primary +  Qranic_Education +  Unknown_Education +  Retired +  Student +  Unemployed +  Unknown_Employment +  Other_Marital_Status +  Single +  Unknown_Marital_Status +  Year_enrolled_in_Care +  Surge +  COVID +  February +  March +  April +  May +  June +  July +  August +  September +  October +  November +  December +  Capital_city +  Abia +  Adamawa +  Anambra +  Bauchi +  Bayelsa +  Benue +  Borno +  Cross_River +  Delta +  Ebonyi +  Edo +  Ekiti +  Enugu +  Federal_Capital_Territory +  Gombe +  Imo +  Jigawa +  Kaduna +  Kano +  Katsina +  Kebbi +  Kogi +  Kwara +  Lagos +  Nasarawa +  Niger +  Ogun +  Ondo +  Osun +  Oyo +  Plateau +  Rivers +  Sokoto +  Taraba +  Yobe +  Zamfara +  Age18_24 +  Age35_44 +  Age45_54 +  Age55_64 +  Aage65ge +  NNRTI +  Undocumented_ART +  PI +  WHO_missing +  WHO_II +  WHO_III +  WHO_IV

The year of enrollment in the study was associated with the outcome, with higher hazards for more recent enrollments (aHR =  1.21, 95% CI: 1.20-1.21, p <  0.001). Being a Surge Enrollee (aHR =  1.14, 95% CI: 1.12-1.15, p <  0.001) and being a COVID Enrollee (aHR =  0.17, 95% CI: 0.17-0.17, p <  0.001) remained significantly associated with different hazards of the outcome compared with being a pre Surge or Pre COVID enrollee, respectively. Residing in an capital city showed an association with the outcome, with individuals from capital cities having a 1.06 (95% CI: 1.05-1.07) times higher risk of being interrupted in treatment within the same time period than those from non capital cities (p <  0.001).

## Discussion

In this study, we investigated the predictors of treatment interruption using data from the national HIV treatment program. We identified sets of predictors of first interruption in treatment using the logistic regression and these to be consistent in predicting time to first interruption including sex, anchor drug in ART regimen, recorded HIV viral load, recorded CD4 cell count, WHO clinical staging, functional status, last measured weight, highest education attained, occupation, marital status, year enrolled in care, pre and post surge, pre and post-COVID and residing in a state capital, Lagos, FCT (capital city) versus other locations (non capital city). Age grouping was the only variable that was predictive only for time to first interruption but not for having a first interruption.

Logistic regression captured some predictors of IIT, including female sex. In the 2019 study by Aliyu et al [17] 2021 study by Tomescu et al [16], the 2022 study by Hakizayezu et al [13], and a 2020 study by Abebe et al [21] sex was found to be predictive of interruptions in care; however, in all of these studies, the men were at a higher risk of interruption or its proxy such as loss to follow up. Our findings may differ from those of prior studies because we were restricted to those who interrupted but re-engaged in care. It may be that men are more likely to discontinue care completely and that women interrupt and return. This then requires more intense follow-up or development of new strategies to keep men in care. PLHIV at WHO clinical stage I for AIDS had the highest odds of experiencing IIT (this is also at variance with the Abebe et al study where Stages I and II combined were demonstrated to have a lower risk of IIT [21]). PLHIV with the functional status of working were most likely to experience IIT (the reverse was seen in the Abebe et al study with ambulatory or bedridden at greater risk [21]). This pattern may suggest that people who feel they are healthy are more likely to interrupt and consequently drop out of care hence the need for educating PLHIV that as their status improves they need to remain in care as well as encouraging those without symptoms to remain in care and educating them on the risks of interruption. Students when compared with the employed were at higher risk of interruption. We found that being retired or unemployed was more protective than employment. This is counterintuitive as we would expect financial stability to be related to improved retention [21,22]. The enrollment year variable was suggestive of the longer people were in care the more likely they were to experience an interruption event. This is consistent with the finding in the Aliyu et al study where duration on ART was found to be related to the odds of loss to follow up with the odds of remaining in care improving with the longer duration of treatment [17]. PLHIV enrolled after the commencement of the surge effort in 2019 were at a higher risk of IIT. Enrollment after the initiation of the surge initiative was linked to a higher risk of interruption which begs the question of the quality of follow-up after aggressive identification of cases and enrollment into care. PLHIV enrolled before March 2020 were at a higher risk of experiencing IIT compared to those enrolled before. People living outside of state capitals, the federal capital territory, and Lagos State were more likely to experience at least one interruption event, this mimics the pattern seen when comparing urban to rural dwellings in a prior study [21]. The Hakizayezu et al study identified other predictors, including a prior history of treatment interruption (which we could not explore as were looking at first interruption) and good perception toward the whole-of-life treatment [13]. Aliyu et al identified additional predictors including HIV viral load indication and result as well as age [17] (which was not predictive in this study or in the Tomescu et al study [16]), and regimen at the start of ART. Other identified predictors in the Tomescu et al study included geo-political zone, regimen line, and viral load suppression [16].

The findings with the proportional hazards regression were in some cases as expected as opposed to those of the logistic regression. Sex was not predictive of time to treatment interruption unlike in predicting being interrupted. The instantaneous risk worsened as the WHO clinical stage worsened in our study when compared to stage I. Age groups, which was not predictive of being interrupted in treatment, were predictive of time to interruption with a trend towards a lower risk with increasing age. This was consistent with the APIN study where younger age was found to be predictive of people under 30 years of age at the greatest risk of LTFU [14]. We found that the ART class of medication was predictive with NNRTI, and PI having greater instantaneous risk than INSTI. This is contrary to the 2014 Meloni et al study that did not find starting an ART regimen to be predictive [14]. Being married had a higher instantaneous risk of IIT than being single contrary to the reverse that was found in the Meloni et al study in Nigeira [14]. Having HIV viral load measured posed a greater instantaneous risk in the case of logistic regression however the reverse was found for having measured and recorded CD4 cell count. In most of the time periods covered in the dataset, CD4 was not required for ART initiation and was run for cause (regimen choice, etc.). In addition, HIV viral load was not run at baseline and, as such, likely is a pointer to engagement in care. Having Q’ranic or tertiary education were both demonstrated to be protective of IIT when compared to secondary education, and this is consistent with the Meloni et al study where tertiary education was found to be protective compared to secondary education [14]. For the year of enrolment, we found the instantaneous risk increases as the years become more recent (moving from 2015 to 2021), again in contrast to the findings with the logistic model. The Meloni et al study did not find a significant difference in the year of ART initiation [14]. However consistent with logistic regression we find that people who enrolled after the commencement of the surge effort are more likely to experience interruption while those enrolled after the COVID lockdown of March 2020 are protected. PLHIV enrolled after the outbreak demonstrated a lower risk of interruption, potentially due to increased emphasis on healthcare access and continuity during the pandemic. However, individuals enrolled before the pandemic experienced a higher risk of interruption, indicating the need for targeted interventions to mitigate the effects of external factors on HIV care. Capital city dwellers (in state capitals, FCT, or Lagos) have a higher instantaneous risk of interruption consistent with the logistic regression and other studies [21]. In the Meloni et al study, additional predictors identified included CD4 cell count [11,14] while in the NCH study baseline CD4 count was found to be a predictor [11]. Across the two approaches used, we found some predictors to have intersected including measured and recorded HIV viral load, surge enrollment, and COVID enrollment. Others that were predictive but in different directions included sex, measured and recorded CD4 count, marital status, and WHO clinical stage.

The rate of missed appointments increased as the duration of care progressed, emphasizing the need for interventions to improve appointment adherence, not only in the early stages of care but also as treatment fatigue potentially sets in [23]. The 25-34 age group constituted the largest proportion of the population, indicating the importance of targeted interventions and support for this age cohort who are also at higher risk of interruption in treatment. Overall, the findings of this study contribute to our understanding of care interruptions. A huge takeaway from this study is the potential for differentiated service delivery, a person-centric approach to care and treatment in HIV in Nigeria and sub-Saharan Africa as we see multiple factors interacting to determine the outcomes of interruption in treatment and progression to loss to follow-up.

### Limitations of the Study

Our study design was retrospective, limiting our control over data collection and the availability of variables of interest including access to care and type of healthcare facility. In addition, we did not include certain variables because of the degree of missingness such as CD4 cell count, HIV viral load (both of which were dichotomized to included or missing), pregnancy status, comorbidities including tuberculosis, and other potential confounders. The findings of this study are dependent on the accuracy and completeness of the recorded data. Potential limitations such as missing or incomplete data, coding errors, or inaccuracies in medical records could affect the validity of the results.

## Conclusion

To reduce the risk of IIT, it is crucial to implement preemptive, targeted interventions. We emphasize the need for tailored approaches that address the unique needs of PLHIV in Nigeria. Interventions should focus on those with combined risk factors and include education, counseling, supportive services, and monitoring. There should be continued education, and improvement on education strategies for PLHIV on the importance of adhering to treatment and the risks of IIT augmented with appointment reminders and rescheduling especially as they improve in metrics such as weight, physical activity, WHO classification, or even CD4 and HIV viral load counts [24].
